# Genome-Based In Silico Analysis of the Structural and Functional Characteristics of the Type Three Secretion System (T3SS) and Core Effector Proteins in Enteropathogenic *Escherichia coli* (EPEC) Strains Isolated from Food-Producing Animals and Products of Animal Origin

**DOI:** 10.3390/pathogens14111099

**Published:** 2025-10-29

**Authors:** Refiloe Malesa, Rian Pierneef, Thendo Mafuna, Kudakwashe Magwedere, Emmanuel Seakamela, Itumeleng Matle

**Affiliations:** 1 Bacteriology Division, Agricultural Research Council-Onderstepoort Veterinary Research, Onderstepoort 0110, South Africa; seakamelae@arc.agric.za; 2 Department of Agriculture and Animal Health, Science Campus, University of South Africa, Florida 1709, South Africa; ematlei@unisa.ac.za; 3 Department of Biochemistry, Genetics and Microbiology, University of Pretoria, Pretoria 0001, South Africa; rian.pierneef@up.ac.za; 4 Centre for Bioinformatics and Computational Biology, University of Pretoria, Pretoria 0001, South Africa; 5 Department of Biochemistry, University of Johannesburg, Auckland Park, Johannesburg 2001, South Africa; tmafuna@uj.ac.za; 6 Center on Emerging Infectious Diseases, Boston University, 111 Cummington Mall, Suite 140, Boston, MA 02215, USA; gwedas@yahoo.co.uk

**Keywords:** Enteropathogenic *E. coli*, T3SS, protein domains, transmembrane domains, locus of enterocyte effacement, protein–protein interaction, protein structure

## Abstract

Enteropathogenic *Escherichia coli* (EPEC) is a significant diarrheagenic pathotype responsible for severe gastrointestinal infections, particularly in vulnerable populations. The aim of this study is to utilize genome-based in silico analysis to study the structural and functional characteristics of the Type III Secretion System (T3SS) and its core effector proteins in EPEC strains. Representative proteins were selected, with particular interest placed on EscV and EscD as major parts of the export apparatus and the basal body, while the EspA effector protein forms the filamentous structure. Several in silico-based techniques were employed, revealing key structural proteins, core effectors, and adhesion-related proteins among the sequenced isolates. Of the 27 isolates analyzed, only 3 (11%) were found to carry LEE-encoded proteins associated with T3SS structural components (escV, escN, escD, and escU) and core effector proteins (espA, espD, espG, and eae). Structural predictions and Ramachandran plot validations suggested stability and potential functional conservation of T3SS proteins, with EscV and EspA selected for detailed 3D structural modelling. Insights into transmembrane domains, protein–protein interaction, and secondary structures were obtained, providing a comprehensive understanding of T3SS assembly and function. These findings suggest that the T3SS in EPEC consists of stable proteins that enable the system to remain functional. The structural and functional properties of the LEE genes encoding the T3SS in the EPEC pathotype represent promising targets for developing virulence blockers to disrupt the pathogenesis of a broad range of bacteria. This study is the first to report EPEC strains with functional T3SS in South Africa, emphasizing the importance of continued surveillance and molecular characterization of EPEC strains. The findings contribute to the development of targeted interventions to mitigate foodborne infections and improve public health.

## 1. Introduction

A 40% reduction in the global average incidence of foodborne diarrhoeal diseases, calculated per 100,000 population, is one of the key indicators outlined in the World Health Organization (WHO) Global Strategy for Food Safety. This ambitious target, set for achievement by 2030, reflects a global commitment to improving public health by reducing the burden of foodborne diseases. The reduction analysis specifically considers the incidence of diarrhoeal diseases caused by five major pathogens: Shiga toxin-producing *Escherichia coli* (STEC), *Campylobacter* spp., Enteropathogenic *E. coli* (EPEC), Enterotoxigenic *E. coli* (ETEC), and Non-typhoidal *Salmonella enterica*. These pathogens were selected due to their significant contribution to the global burden of foodborne illness, as highlighted in the WHO’s 2021 report.

Enteropathogenic *Escherichia coli* (EPEC) is a significant diarrheagenic pathotype known for causing severe infant diarrheal outbreaks, particularly in developing countries where public health infrastructure may be limited [1,2]. EPEC infections are typically transmitted to humans through the consumption of contaminated food, often because of inadequate sanitation and hygiene practices in food production and handling [3]. This mode of transmission poses a substantial risk to public health, especially in regions prone to diarrheal disease outbreaks.

Enteropathogenic *Escherichia coli* (EPEC) has been isolated from a diverse array of food sources, including meat, seafood, vegetables, fruits, and dairy products, underscoring the widespread nature of its contamination potential [4,5,6,7,8]. Contaminated animal products represent a notable risk, given their frequent consumption, especially among vulnerable populations [9]. Animals, particularly livestock, can act as reservoirs for the EPEC, thereby facilitating transmission to humans. As a result, surveillance of EPEC in animal populations and animal-derived products becomes essential for public health risk mitigation.

Given EPEC’s pathogenicity and its health implications, there is a strong need for ongoing surveillance of its transmission pathways, virulence factors, and strains, as well as understanding the mechanisms by which these strains contribute to disease outbreaks. Such efforts are critical for developing effective prevention strategies and safeguarding public health from EPEC-related infections [10,11]. Upon attachment to the host intestinal epithelium, EPEC employs a type III secretion system (T3SS) to inject effector proteins into the host cells. The T3SS is known as a transport apparatus primarily used by Gram-negative bacteria to insert effector proteins into host cytoplasmic cells. This secretion system allows bacteria to manipulate host cellular processes, helping them establish infections, evade the immune response, and promote their own survival [12,13]. The effector proteins delivered by the T3SS can disrupt cellular signalling, induce inflammation, and facilitate bacterial entry into host cells [14].

Typical EPEC has the locus of enterocyte effacement (LEE) region, which is a 35.6 kb pathogenicity island that encodes the T3SS [15,16,17]. The LEE pathogenicity region is categorized into five gene clusters, named LEE1 to LEE5 [16], which harbour 41 genes that encode structural components of the T3SS, including six translocated effectors, regulators, and chaperones [13]. The esc genes found in the inner and outer membranes of the T3SS are encoded by the LEE1 to LEE3 gene clusters and contribute to the formation of the core structures of the system. The LEE4 consists of translocator proteins EspD and EspB, which are responsible for the assembly of the T3SS. The Tir effector is encoded by LEE5 [17]. Effector proteins encoded by the T3SS are found within and outside the LEE, alongside non-LEE effectors [12].

Non-LEE effector proteins play a significant role in enhancing inflammation and inducing cell death within the epithelium [12]. Non-LEE encoded T3SS effectors, such as Cif, NleA to NleH, EspL, EspJ, and EspO, perform distinct functions within the host cellular system to facilitate infection [17]. The presence of these non-LEE effector proteins varies among different EPEC serotypes, with some effectors being absent in certain strains [18].

The T3SS comprises diversified proteins with varied functions. For instance, escU, escV, escR, escT, and escS form the export apparatus, while escN, escL, and escO comprise the cytosolic ATPase complex. Additionally, the effector protein espA forms a filament, and espB and espD create the translocation pore. The assembly and translocation of effectors within the T3SS rely on these proteins [19]. Among the LEE-encoded proteins, escV contributes to the structural stability of the T3SS, escD forms part of the injectisome, allowing effectors to pass through, and escU is involved in the secretion of translocator proteins. Meanwhile, escN hydrolyzes ATP to provide the energy needed for effector secretion [20].

The export apparatus is assembled from five highly conserved membrane proteins: escR, escS, escT, escU, and escV. EscV consists of two large domains: a cytoplasmic domain at the C-terminal end and an N-terminal pathway with seven to eight transmembrane domains (TMDs). The recruitment of T3SS substrates, chaperones, and proteins from the “gatekeeper” family to the T3SS apparatus is linked to escV and its homologs in both pathogenic and flagellar T3SS [20,21,22,23]. EscD is a member of the single-span transmembrane proteins in the EscD/PrgH/YscD family, characterized by a larger C-terminal periplasmic domain and a smaller N-terminal cytoplasmic region [24,25,26].

The use of genome-based analysis has allowed analysis of various gene clusters within the pathogenic islands found in bacteria that are responsible for the assembly and functioning of the T3SS [27]. Structural prediction of the T3SS using in silico-based techniques, such as the Swiss-model, enables the construction of three-dimensional structures of proteins using their amino acid sequences; with these tools, comparative analysis of various proteins within the T3SS can be employed [28,29]. This study employed in silico genome-based approaches to predict the functional characteristics of T3SS structural proteins as well as core effector proteins found in EPEC strains isolated from food-producing animals and products of animal origin. The study specifically focused on core proteins, EscV and EscD only, as they play a major role in exporting proteins and formation of the basal body, while the EspA protein was selected due to its key role in the assembly of the filament and an effector protein that interacts directly with the host.

## 2. Materials and Methods

### 2.1. Bacterial Strains

A total of 27 previously identified EPEC strains [30] isolated from food-producing animals and products of animal origin in South Africa and imported meat products from North America and Europe were utilized in the current study (Table 1). These isolates were selected to represent a range of geographic regions, and the sample sources were selected to ensure that both hosts and environments where *E. coli* occurs are represented. DNA extraction, data pre-processing, and whole genome sequencing of the isolates were performed as previously described by Malesa and team [30].

### 2.2. Determination of LEE Effector Proteins

To screen for locus of enterocyte effacement (LEE), a query coverage of 85% and 95% identity was used as criteria for selecting T3SS genes for the study. DNA sequences were then analyzed using ORFFinder (https://www.ncbi.nlm.nih.gov/orffinder?tdsourcetag=s_pcqq_aiomsg, accessed on 10 September 2024) to confirm the presence of open reading frames.

The Virulence Factor Database (VFDB) [31] was used to identify the virulence factors associated with our isolates. Based on the VFDB results, various contigs were analyzed using MEGA v11 to pinpoint the locations where these genes were harboured. Nucleotide sequences of each contig were selected and translated into protein sequences using MEGA v11, focusing on a subset of conserved proteins included in the study, escV, escD, escU, escN, espA, espD, espG, and eae.

### 2.3. Functional Annotation of Proteins

Further analysis involved examining the protein sequences using the Simple Modular Architecture Research Tool (SMART) based on Hidden Markov Models to identify and annotate signalling domains [32]. The Genomic SMART mode (https://smart.embl.de/smart/change_mode.cgi, accessed on 10 September 2024) was employed for protein analysis in this study, with searches conducted using default settings. Conserved protein domains were verified through the NCBI Conserved Domain Database (CDD) [33], with input protein sequences processed under default conditions.

### 2.4. Validation of SMART

To assess sequence similarities for each protein, a BLASTp search was conducted for each protein sequence [32]. The query protein sequences were analyzed using default settings, with results considered valid if they exhibited a query coverage of 90% and a percentage identity exceeding 95%.

### 2.5. Analysis of Physiochemical Properties

To assess protein stability and composition, the physicochemical properties of each protein were determined using the ProtParam tool on Expasy (https://web.expasy.org/protparam/, accessed on 12 September 2024). Each protein sequence was analyzed under default parameters.

### 2.6. Protein–Protein Interactions

The STRING database (https://string-db.org/, accessed on 12 September 2024) was used to construct a network of interactions between distinct known and predicted proteins. The analysis was performed using a full STRING network, with network edges adjusted to confidence mode, a minimum interaction score of 0.4, and a maximum of 20 interactions.

### 2.7. Determination of Transmembrane Domains

Transmembrane domains (TMDs) were identified using the TMHMM v2.0 tool (https://services.healthtech.dtu.dk/services/TMHMM-2.0/, accessed on 12 September 2024) for predicting transmembrane protein helices, with confirmation of TMDs performed using the PHOBIUS tool (https://phobius.sbc.su.se/, accessed on 10 September 2024). The topology of the sequences was then determined using Protter (https://wlab.ethz.ch/protter/start/, accessed on 10 September 2024). For each tool, protein sequences were input and analyzed using default settings.

### 2.8. Protein Structure Prediction

Protein structures of the components of the T3SS were predicted using Swiss-Model (https://swissmodel.expasy.org/, accessed on 14 September 2024). The target sequence was submitted to the server, which automatically searched for templates that were selected based on sequence identity. Model quality was assessed using the Ramachandran plot and MolProbity server version 4.2. To enhance the robustness and reliability of the predictions, additional tools were employed. The SOPMA tool (https://npsa-prabi.ibcp.fr/cgi-bin/npsa_automat.pl?page=/NPSA/npsa_sopma.html, accessed on 14 September 2024) was used for accurate prediction of the secondary structure of each protein.

### 2.9. Multiple Sequence Alignment

Multiple sequence alignment of the three (escV, escD, espA) proteins analyzed in this study was aligned with homologous sequences retrieved from the NCBI database based on sequence similarities. The COBALT multiple alignment tool from NCBI was used for alignment (https://www.ncbi.nlm.nih.gov/tools/cobalt/re_cobalt.cgi, accessed on 29 September 2025) using default parameters. The alignment was visualized using the Jalview tool version 2.11.5 [34], 2004).

## 3. Results

### 3.1. Analysis of Virulence Genes

Upon analyzing the VFDB results, various genes encoding T3SS proteins were identified. Notably, only 3 isolates (SAMN41920865, SAMN41920868, and SAMN41920872) carried LEE proteins associated with T3SS structural properties (Table S1); however, proteins from LEE1 through LEE5 were detected.

### 3.2. Determination of Protein Domains

Several LEE-encoding T3SS proteins were analyzed using SMART and subsequently confirmed with the CDD tool. Genes with a significant impact on T3SS function were selected and categorized based on their roles. The detected proteins included those associated with structural formation, such as escV, escN, escD, and escU; core effector proteins related to pathogenicity, including espA, espD, and espB; and genes responsible for adhesion and attachment, such as eae (Table 2). According to the SMART analysis, fifteen protein domains related to the T3SS were identified, with the majority classified using the Protein Families Database (Pfam). Of the eleven proteins analyzed, the nleB1protein could not be linked to a specific protein domain. Each protein was associated with a single protein domain, except for the eae protein, which exhibited at least six distinct domains (Table 2).

### 3.3. Analysis of SMART Validation

The selected proteins showed high similarity to known *E. coli* proteins in the NCBI BLASTp database. Each protein analyzed displayed 100% query coverage and over 99% sequence identity with existing protein sequences (Table 3).

### 3.4. Physiochemical Properties of Encoded T3SS Proteins

Analysis of the LEE encoding proteins revealed significant variability in protein lengths. The proteins eae, escV, escN, and escD were among the longest (insert the length), while espA and escU were considerably shorter (insert the length). This variation in length correlated with molecular weight: eae, with 939 amino acids, had a molecular weight of 101,684.77 Da, whereas espA, with 192 amino acids, had a molecular weight of 20,534.02 Da. The Grand Average of Hydropathicity (GRAVY) results indicated that escU, escN, and espG are hydrophobic proteins, whereas eae, espA, espD, and escV are hydrophilic. The instability index further revealed that escU, escN, and eae are relatively stable proteins, with indices below 40, while espD and escV showed indices close to the instability threshold, but these proteins are still below 40 and are considered to be relatively stable (Table S2). All non-LEE encoding genes were associated with proteins classified as hydrophilic based on their GRAVY results. These proteins exhibited variability in length and molecular weight, but their aliphatic indices were similar, ranging from 82 to 88.

### 3.5. Analysis of Protein–Protein Interactions

**Figure 1:**
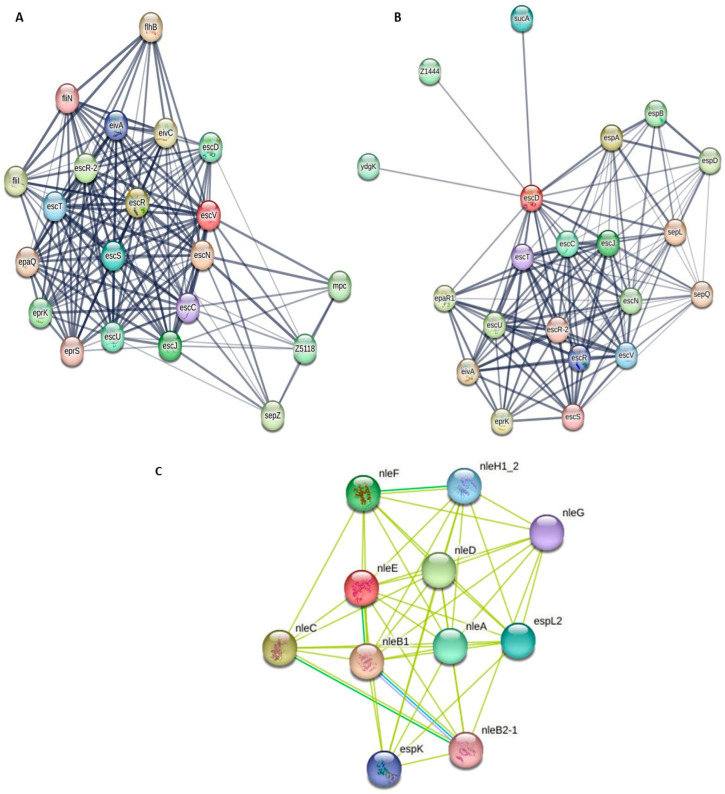
Protein–protein interaction network was generated using the STRING database. (**A**) represents the escV, escN proteins, (**B**) represents the escD, escU, espA, espD, (**C**) represents the non-LEE proteins, nleE, nleB1, nleG. The grey and yellow lines in between the nodes represent the interactions between proteins.

Protein interactions were analyzed using the STRING database (Figure 1). All proteins studied showed high identity to the *E. coli* O157 strain (EDL933) with a taxonomic identifier of 155,864. The protein networks for both LEE and non-LEE proteins demonstrated that they are associated with other known bacterial secretion proteins. The T3SS complex was found to include proteins such as Flil, EprK, EprS, and EscJ. Proton-transporting ATPase activity is associated with Flil, EivC, and EscN. Most of these proteins are involved in biological processes such as protein secretion, protein transport, and other cellular functions. Specifically, the T3SS complex is present in EscC, EscJ, and EscN.

### 3.6. Analysis of Transmembrane Domains (TMD)

**Figure 2:**
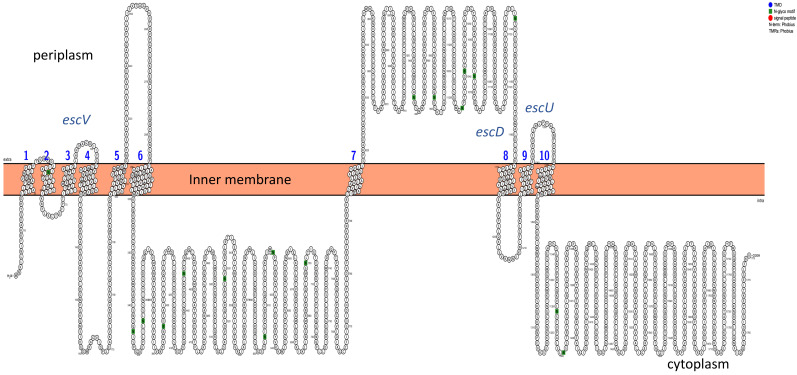
Predicted topology of T3SS proteins that are composed of transmembrane domains, structure determined using Protter. Structural protein escV consists of seven (1–7) TMDs, protein escD consists of one (8) TMD, protein escU consists of two (9–10). Inner membrane approximated with an orange rectangle. Letter N in green represents the N-glyco-motif sequences.

**Figure 3:**
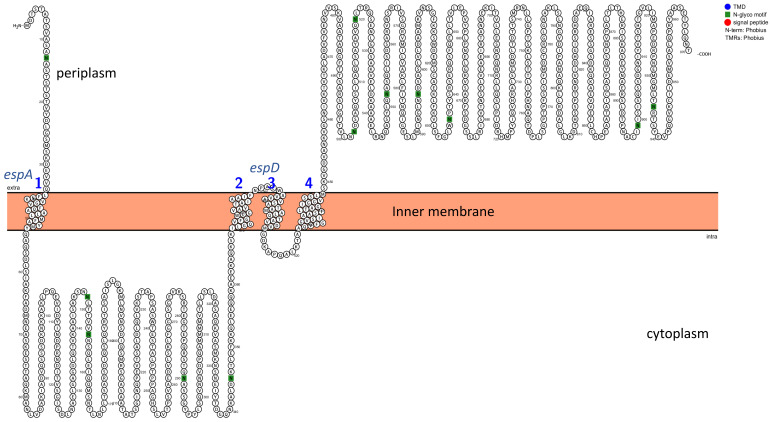
Predicted topology of T3SS Core effector protein espA, consisting of one (1) TMD and protein espD, consisting of three (2–4) TMDs. Inner membrane approximated with an orange rectangle. Letter N in green represents the N-glyco-motif sequences.

Transmembrane domains (TMDs) were analyzed using Phobius, and the structural topology was determined with Protter. The structural protein escV was found to have seven TMDs, escU had two TMDs, and escD had one TMD (Figure 2 and Figure S1). Additionally, N-glyco motifs were identified in these sequences, while signal peptides were absent. Analysis of the T3SS core effector proteins revealed that the espA protein had one TMD, and espD had three TMDs (Figure 3). Non-LEE proteins were found not to contain any TMDs.

### 3.7. Predicted Protein Structures

**Figure 4:**
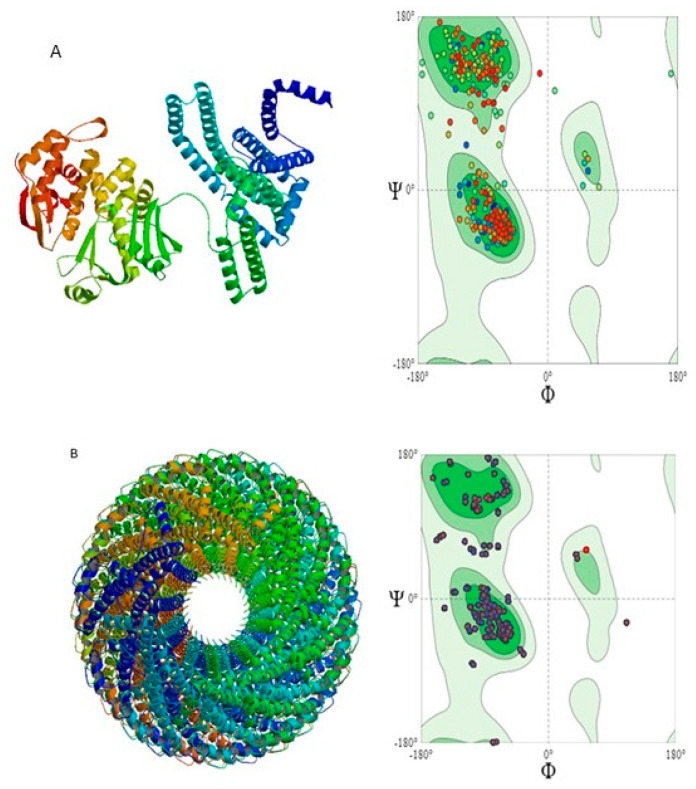
Three-dimensional structure of proteins and their Ramachandran plots, (**A**) escV protein, 96.14% of amino acids are on the favoured region (beta-sheet), 1.19% amino acids are outliers in this region; (**B**) espA protein with 92.36% amino acid residues on the favoured region, with 0.64% outliers.

**Figure 5:**
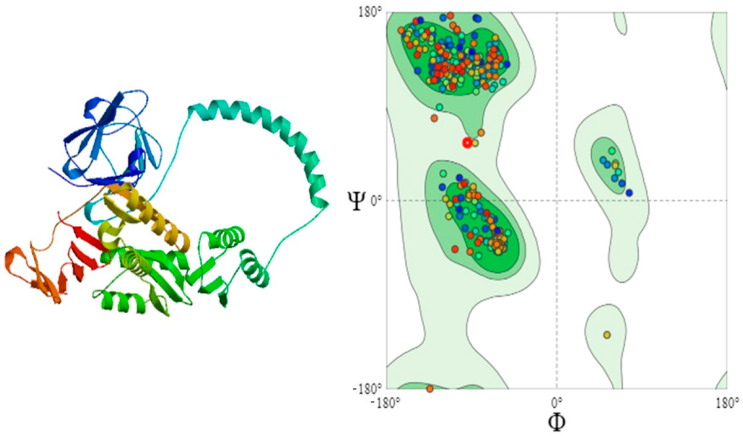
Three-dimensional structure of protein escD and its Ramachandran plot, 94.9% of amino acids are in the favoured region (beta-sheet), while 1.81% amino acids are outliers.

Three proteins were selected for three-dimensional structural analysis: escV and escD to represent structural proteins and espA to represent core effector proteins (Figure 4 and Figure 5). Based on sequence identity, coverage, and Ramachandran plot analysis, the most appropriate models were chosen. The template selected for escV was A0A3I8FV28.1A, which showed a sequence identity of 78.22% to the EscV/YscV/HrcV family T3SS export apparatus protein, and the structure consisted of a single subunit (monomer). The selected model for espA was 7khw.1.J, a translocon protein with 88.54% sequence identity, featuring an oligomeric state of homo-50-mer. For the escD protein, the most appropriate template selected was B1EHF2.1.A, which had 95.07% sequence identity.

### 3.8. Quality Assessment of 3D-Structures

The Ramachandran plot quality assessment for the escV and espA proteins yielded MolProbity scores of 1.38 and 1.35, respectively. The majority of residues observed in the Ramachandran plot were well-folded, with 96.14% of residues for escV and 92.36% for espA (Table 4).

### 3.9. Secondary Structure of the Proteins

The analysis of the secondary structure of the proteins in terms of alpha-helix, beta-sheet, and coil states (Table 5) was conducted. Protein escV, consisting of 675 amino acids, exhibited 52.59% alpha-helix, 17.48% extended strand, 5.48% beta-turn, and 24.44% random coil. Protein espA, with 192 amino acids, had 68.75% in alpha-helix, 2.08% in extended strand, no beta-turn, and 29.17% in random coil.

### 3.10. Conservation of Proteins

Sequence alignment revealed high conservation across protein sequences included in this study. Conserved motifs were observed in various positions 430–490 (Figure S2). The C-terminal region also exhibited conserved segments, which indicates structural stability.

## 4. Discussion

To thoroughly analyze the various components of the type three secretion system detected in EPEC strains in South Africa, EscV, EscD, and EspA were intentionally selected. The EscV protein was selected to represent the inner membrane export apparatus, the EscD protein for its importance in maintaining the structure of the system, and EspA as the needle component, which is the extracellular filament of the system [35].

Enteropathogenic *E. coli* is one of the most important causes of diarrhoeal disease in both humans and animals, often resulting in high mortality rates in children, especially in developing countries [36]. Typical EPEC strains often consist of the locus of enterocyte effacement (LEE), which is a region used for attaching and effacing to colonize the host’s intestines. This region encodes for the T3SS [37]. This study was carried out to predict the structural and functional characteristics of the T3SS in EPEC strains using various in silico techniques. To the best of our knowledge, in South Africa, there is currently no published data that investigates the T3SS in EPEC strains.

In other countries, the occurrence of the T3SS has been identified in EPEC strains from various sources, and these isolates have been found to possess various virulence genes that are crucial for EPEC’s pathogenicity. In African countries, there are very few studies that are sequenced, which are focused on characterizing EPEC strains. In a study conducted in Nigeria, it was found that one EPEC isolate contained a hemolysin gene, *ehx*, which is commonly found in EHEC pathotypes, and the presence of non-LEE genes was also detected [38].

A study was conducted in 2023, which analyzed three diarrheagenic *E. coli* pathotypes (EAEC, EPEC, and STEC) in three African countries, Gambia, Kenya, and Mali, over a period of three years. This study highlighted atypical EPEC as the second most common pathotype to occur, which often results in high mortalities [39]. In Zambia, Kenya, Mozambique, Rwanda, and Sudan, EPEC has been reported as the most prevalent pathotype [40,41,42].

Moreover, a systematic review of *E. coli* virulence and antibiotic resistance profiles in various African countries revealed that in Africa, antibiotics are abused, and this has led to widespread resistance. Also, virulence determinants of *E. coli*, such as *eae*, *stx1*, *stx2*, *ast*, *fliC07*, *papC*, and *eagg* virulence genes have been found to occur across various *E. coli* matrices. This observation was found to be evident in South Africa and Nigeria, as they tend to test *E. coli* on various matrices [43].

Protein domains play a crucial role in the functioning of the Type III Secretion System (T3SS). The flagellum/hypersensitive response/invasion protein export pore (FHIPEP) domain, which is part of the conserved export protein domains, has not been fully characterized, but it is thought to play a role in sorting substrates into the pore of the export apparatus to aid in the delivery of effector proteins [44]. In proteobacteria, the Yop-YscD_ppl domain is part of the periplasmic domain of Yop proteins and forms part of the inner membrane of the injectisome [45]. Bacterial transmembrane domains, which are essential for bacteria to move along host cellular membranes, contain proteins that target the host, playing a key role in infection [46].

The effector proteins encoded by the Locus of Enterocyte Effacement (LEE) pathogenicity island in T3SS include espF, espG, espH, espZ, and map, along with translocator proteins espA, espB, and espD [47,48]. As one of the largest export apparatus proteins, escV, is responsible for controlling the export gate of the T3SS, while escD forms part of the inner membrane of the basal body and plays a critical role in secretion regulation within the inner membrane [49]. Additionally, the ATPase activity of escN drives its interactions with T3SS effectors and chaperones [50].

EscV consists of 675 amino acids and has a molecular weight of 75,150.08 Da, while escD has 406 amino acids and a molecular weight of 45,316.26 Da. The aliphatic index, calculated based on the percentage of alanine, valine, isoleucine, and leucine, was high for both proteins, indicating increased thermostability. Both proteins were also determined to be stable, with instability index values below 40 (escV 39.61, escD 39.83), suggesting they are less prone to denaturation under physiological conditions [51].

The theoretical isoelectric point (pI) of the proteins ranged from 4 to 10, suggesting electrical neutrality under normal conditions. The Grand Average of Hydropathicity (GRAVY) for escV was positive (0.355), indicating hydrophobic characteristics, while escD had a negative GRAVY value (−0.087), suggesting it contains hydrophilic amino acids [52]. Though hydrophobicity is only one factor, it plays a key role in understanding protein solubility and interactions [53].

Protein interactions for escV and escD were analyzed using the STRING database, with *E. coli* O157 EDL933, a highly pathogenic strain, selected as a reference. The escV protein shared 97.9% identity with the reference strain, while escD shared 96.8%. Both proteins were found to interact with other related T3SS proteins such as escT, escR, escS, and escU, which form part of the conserved export apparatus between the flagellar system and the injectisome [14,54]. EscV forms a pore in the inner membrane through which effectors are translocated, while escD is part of the base structure of the injectisome, interacting with escC and escJ proteins to maintain its structural integrity [14].

The conserved domains of these proteins were identified using the Conserved Domain Database (CDD), while the transmembrane domains (TMD) were predicted with TMHMM v2.0. EscV was found to have seven TMDs, each with varying residues. This protein plays a critical role in recruiting T3SS chaperones, substrates, and other proteins, facilitating the assembly and function of the T3SS [20]. Both proteins’ 3D models were validated using Ramachandran plots, with over 90% of the residues falling within favoured regions, indicating high model quality [55]. The MolProbity score for these structures was <1.8, further confirming their reliability [56].

Secondary structures, including beta strands, beta turns, alpha helices, and random coils, were predicted using the SOPMA tool. These structural elements are crucial for protein stability, serving as binding sites for other molecules and influencing protein function [57]. This detailed characterization of escV and escD enhances our understanding of their role in the T3SS and provides insights into the molecular mechanisms governing their function.

The T3SS comprises various components with diverse functions; these include causing cell death, disrupting the host cytoskeleton, and manipulating cellular activities. Several studies have developed strategies to inhibit activities of the T3SS; some of these include suppressing activities of some effector proteins, which then results in the system being less virulent [35]. For EPEC and EHEC infections, a mouse model using *Citrobacter rodentium* was developed to inhibit the effector protein responsible for adhesion of the T3SS. Although other effector proteins were not inhibited and released into the host, this resulted in minimal infections [35,58]. Studies focused on therapeutics have targeted the use of T3SS since some of the structural and functional characteristics of this system are able to facilitate designs of antibiotics that are mechanism-based; such studies are required to overcome antibiotic resistance [58].

Other inhibitors of the T3SS include the use of chemical compounds such as 2-imino-5-arylidene thiazolidinone, which blocked the system as well as its virulence functions [59,60]. The first ever natural inhibitor used for T3SS was glycolipid caminoside A, which was able to reduce the secretion of EPEC. A compound in body fluids, lactoferrin, was also targeted at the T3SS virulence mechanisms. The disadvantage with some of these natural compounds is their toxicity to eukaryotic cells [60].

Sequence alignment of proteins included in this study showed a high conservation in comparison to other *E. coli* EPEC strains, which contain the same proteins. Apparatus such as the escV and escD in the T3SS structure are known to be well conserved [20]. The espA protein remains conserved due to its helical assembly [61].

## 5. Conclusions

The present study focused on the functional characterization of EPEC strains, specifically investigating the structural and core effector proteins associated with the Type III Secretion System (T3SS) using in silico; therefore, no experimental techniques were employed for this analysis. The findings revealed that the isolates with a functional T3SS identified in this study belong to the EPEC pathotype. Notably, the presence of the Locus of Enterocyte Effacement (LEE) pathogenic island underscores the virulence of these strains, affirming their pathogenic potential. The high-resolution structural analysis, along with robust validation data, indicates that the proteins in question are stable and likely to maintain their functional integrity.

Though there was a lack of experimental studies conducted for this analysis, to the best of our knowledge, this is the first report documenting EPEC strains harbouring a functional T3SS in South Africa. This highlights the importance of continued surveillance and characterization of pathogenic *E. coli* strains within the region. Future research should involve a larger sample size, and experimental validations should be carried out to further support these predictions and to better understand the distribution and epidemiology of EPEC pathotypes carrying functional secretion systems across the country. Expanding the scope of study to include different regions and environmental sources would provide a more comprehensive picture of the pathogenic landscape and contribute to the development of targeted public health interventions.

## Figures and Tables

**Table 1:** Metadata of EPEC strains used in this study (*n* = 27).

Accession No.	Geographic Location	Region	Source of Isolation
SAMN41920845	North America	North America	Raw poultry
SAMN41920846	South Africa	Mpumalanga	Raw poultry
SAMN41920847	Netherlands	Europe	Raw poultry
SAMN41920848	South Africa	Free State	Raw poultry
SAMN41920849	South Africa	Free State	Raw poultry
SAMN41920851	South Africa	Free State	Raw poultry
SAMN41920852	South Africa	Free State	Raw poultry
SAMN41920853	South Africa	Free State	Raw poultry
SAMN41920854	South Africa	Free State	Raw poultry
SAMN41920855	South Africa	Free State	Processed pork
SAMN41920856	South Africa	Free State	Raw lamb
SAMN41920858	South Africa	Free State	Raw pork
SAMN41920860	South Africa	Free State	Raw poultry
SAMN41920861	South Africa	Gauteng	Digestive system
SAMN41920862	South Africa	Gauteng	Processed beef
SAMN41920863	South Africa	Gauteng	Digestive system
SAMN41920865	South Africa	Gauteng	Digestive system
SAMN41920867	South Africa	Gauteng	Digestive system
SAMN41920868	South Africa	Gauteng	Digestive system
SAMN41920869	South Africa	Gauteng	Digestive system
SAMN41920870	Europe	Europe	Raw poultry
SAMN41920871	South Africa	Gauteng	Digestive system
SAMN41920872	South Africa	Gauteng	Digestive system
SAMN41920873	South Africa	Gauteng	Digestive system
SAMN41920876	South Africa	Gauteng	Water
SAMN41920877	South Africa	Limpopo	RTE beef
SAMN41920878	South Africa	Gauteng	Digestive system

Processed pork (Commercially processed pork meat), Digestive system (faecal material), and RTE (Ready-to-eat products).

**Table 2:** Summary of protein domain annotations and their functions.

Proteins	Protein Domain	Position	Function of Each Protein
LEE			
Structural			
escV	Pfam:FHIPEP	26 to 663	It is important for translocation of effector proteins
escD	Pfam:Yop-YscD_ppl	157 to 404	It is involved in assembly and function of the T3SS
escU	Pfam:Bac_export_2	2 to 241	It is important for regulation and stabilization of the apparatus
escN	AAA	169 to 349	It hydrolyses ATP to generate energy required for operation
Pathogenicity			
eae	LysM Pfam: IAT_beta BID_1 BID_1 BID_2 Pfam: Intimin_C	64 to 113 166 to 442 559 to 648 659 to 746 757 to 835 838 to 939	It is important for mediation of attachment of *E. coli* to the intestinal epithelium
Core effectors			
espA	Pfam: EspA	4 to 186	It is important for formation of pilus-like structures that facilitate movement of effectors
espD	Pfam: SseC	117 to 216	It forms the pore in the host membrane that allows effectors to move from bacteria into the host
espG	Pfam: EspG	15 to 397	It disrupts the host cell processes
non-LEE			
cif	Pfam: CIF	81 to 215	Interferes with the host cell cycle
nleE	NleE_fam_methyl	13–168	Alters immunological responses and host cell signalling
nleB1	None		Alters proteins in host cells to affect immune responses

**Table 3:** Results of SMART validation.

Proteins	Query Coverage	E-Value	Percentage Identity	Reference. Accession No.
LEE				
escV	100%	0.0	100%	WP_001037814.1
escD	100%	0.0	99.8%	ELP0616342.1
escU	100%	5 × 10^−102^	99.4%	WP_063856070.1
escN	100%	0.0	99.8%	WP_000622546.1
Eae	100%	0.0	100%	WP_000627895.1
espA	100%	2 × 10^−132^	100%	WP_000381555.1
espD	100%	0.0	100%	WP_000935768.1
espG	100%	0.0	100%	AAC31534.1
non-LEE				
Cif	100%	0.0	100%	WP_000652080.1
nleE	100%	1 × 10^−119^	100%	WP_000609738.1
nleB1	100%	0.0	100%	WP_012578998.1

**Table 4:** Validation summary of the escV and espA protein structures by the MolProbity software v.4.2.

Ramachandran Plot Analysis	escV	espA
MolProbity Score	1.38	1.35
Clash Score	2.41	1.40
Ramachandran Favoured	96.14%	92.36%
Ramachandran Outliers	1.19%	0.64%
Rotamer Outliers	1.35%	0.01%
C-Beta Deviations	0	177
Bad Bonds	0/5384	4/66,450
Bad Angles	24/7285	350/89,750
Twisted Non-Proline	5/652	50/8550

**Table 5:** Elements of the secondary structure.

	escV		espA	
Structure	No. of Residues	Percentage (%)	No. of Residues	Percentage (%)
Alpha helix (Hh)	355	52.59	132	68.75
3_10_ helix (Gg)	0	0.00	0	0.00
Pi helix (Ii)	0	0.00	0	0.00
Beta bridge (Bb)	0	0.00	0	0.00
Extended strand (Ee)	118	17.48	4	2.08
Beta turn (Tt)	37	5.48	0	0.00
Bend region (Ss)	0	0.00	0	0.00
Random coil (Cc)	165	24.44	56	29.17
Ambiguous states	0	0.00	0	0.00
Other states	0	0.00	0	0.00

## Data Availability

The Dataset in this study is available in the NCBI Sequence Read Archive repository with accession number: PRJNA1126085.

## Supplementary Materials

The following supporting information can be downloaded at: https://www.mdpi.com/article/10.3390/pathogens14111099/s1, Table S1: Detected LEE proteins and their grouping. Table S2: Physiochemical properties of LEE encoded proteins. Figure S1: Predicted transmembrane protein domains of LEE and non-LEE genes using Phobius. Figure S2: Multiple sequence alignment of T3SS proteins (espA, escD, escV). The ClustaW scheme was used for colouring residues in this alignment.
