# Supplementary material for: Genome-Based In Silico Analysis of the Structural and Functional Characteristics of the Type Three Secretion System (T3SS) and Core Effector Proteins in Enteropathogenic Escherichia coli (EPEC) Strains Isolated from Food-Producing Animals and Products of Animal Origin
Source: Pathogens. 2025 Oct 29;14(11):1099. doi: 10.3390/pathogens14111099 (PMC12655248; doi:10.3390/pathogens14111099)
Supplement: Supplementary file 1 [file pathogens-14-01099-s001.zip › pathogens-3895167-supplementary.pdf]

SUPPLEMENTARY MATERIAL

Table S1. Detected LEE proteins and their grouping

LEE encoded proteins
espL2, nleB1, nleE, nleA/espI, espM2, espW, cif, nleH1, nleA/espI, nleH2, nleF, espM1, espR1, espX4, espG, escE, cesAB, escL, escR, escS, escT, escU, etgA, cesD, escC, sepD, escJ, escI, cesL, escV, escN, escO, escP, sepQ/escQ, espH, cesF, map, cesT, eae, escD, sepL, espA, espD, cesD2, escF, escG, espF, nleG7', espX5, espX1
**LEE protein groups**
**LEE1**
espG, escE, cesAB, escL, escR, escS, escT, escU
**LEE 2**
cesD, escC, sepD, escJ, escI
**LEE 3**
escV, escN, escO, escP, sepQ/escQ, espH, cesF, map
**LEE 4**
escD, sepL, espA, espD, cesD2, escF, escG, espF,
**LEE 5**
cesT, eae

Table S2. Physiochemical properties of LEE encoded proteins

Physiochemical properties	LEE encoded proteins							
	escU	***escN***	***eae***	***espA***	***espD***	***espG***	*escV*	*escD*
Amino acids	250	446	939	192	380	398	675	406
Molecular weight (Da)	28604.50	48858.45	101684.77	20534.02	39618.76	43905.64	75150.08	45316.26
Aliphatic index	108.32	105.40	76.99	88.49	91.18	88.24	119.81	105.69
Instability index	32.47	35.55	32.60	37.67	37.26	41.70	39.61	39.83
Theoretical pI	9.29	5.99	8.95	4.80	6.65	5.37	5.66	6.96
Total number of negatively charged residues (Asp+Glu)	27	54	78	20	31	46	69	41
Total number of positively charged residues (Arg+Lys)	35	50	89	16	31	35	62	41
Extinction coefficients (all	32890	25120	123665	-	-	24785	51465	49070

pairs of Cys residues form cystine)								
Extinction coefficients (all pairs of Cys residues are reduced)	32890	24870	123540	7450	25440	24410	51340	48820
Grand average of hydropathicity (GRAVY)	0.046	0.020	-0.358	-0.195	-0.025		0.355	-0.087

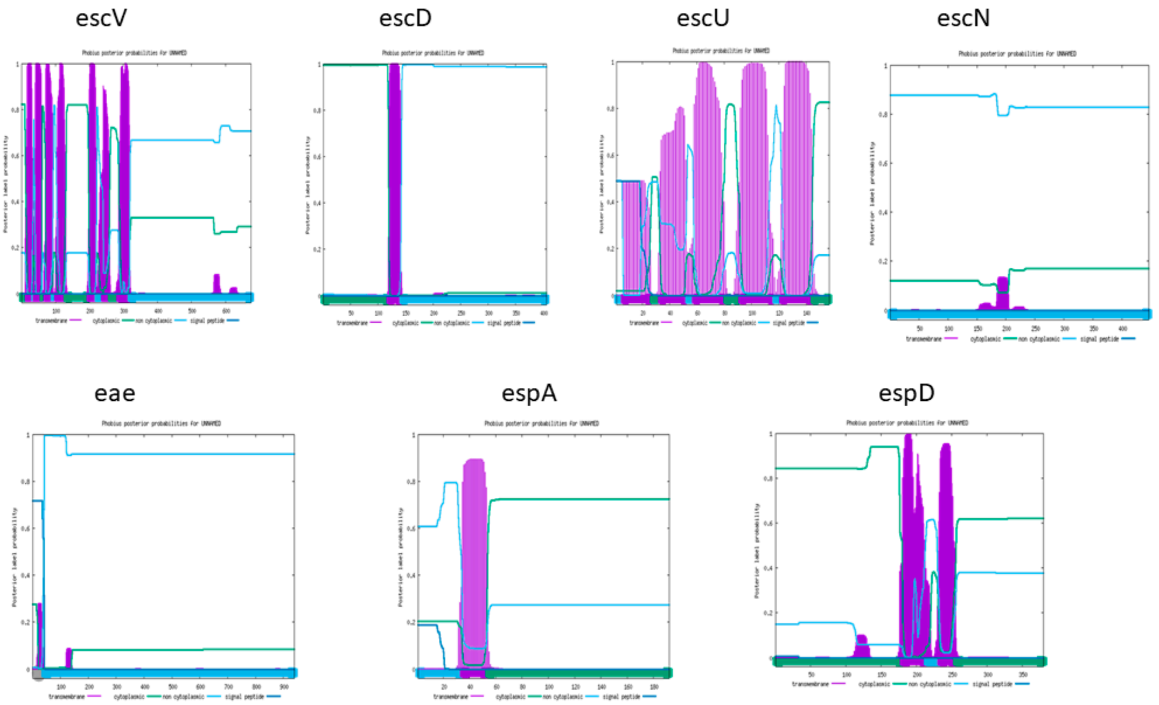

Figure S1. Predicted transmembrane protein domains of LEE genes using Phobius.

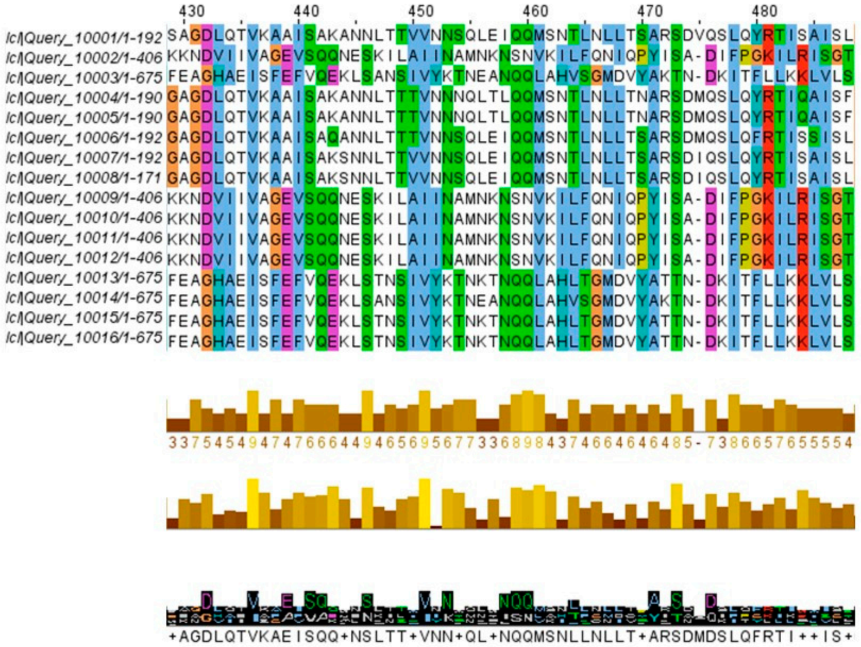

Figure S2. Multiple sequence alignment of T3SS proteins (espA, escD, escV). ClustaW scheme was used for coloring residues in this alignment.
